# Sequence variations in DNA repair gene *XPC *is associated with lung cancer risk in a Chinese population: a case-control study

**DOI:** 10.1186/1471-2407-7-81

**Published:** 2007-05-13

**Authors:** Yun Bai, Liang Xu, Xiaobo Yang, Zhibin Hu, Jing Yuan, Feng Wang, Minhua Shao, Wentao Yuan, Ji Qian, Hongxia Ma, Ying Wang, Hongliang Liu, Weihong Chen, Lin Yang, Guangfu Jing, Xiang Huo, Feng Chen, Yanhong Liu, Li Jin, Qingyi Wei, Wei Huang, Hongbing Shen, Daru Lu, Tangchun Wu

**Affiliations:** 1 Institute of Occupational Medicine and Ministry of Education Key Lab for Environment and Health, School of Public Health, Tongji Medical College, Huazhong University of Science and Technology, Wuhan 430030, PR China; 2 Department of Genetics, Chinese National Human Genome Center at Shanghai, Shanghai 201203, PR China; 3 Department of Epidemiology and Biostatistics, Cancer Research Center of Nanjing Medical University, Nanjing 210029, PR China; 4 State Key Laboratory of Genetic Engineering, School of Life Sciences, Fudan University, Shanghai 200433, PR China; 5 Department of Epidemiology, The University of Texas M. D. Anderson Cancer Center, Houston, TX 77030, USA

## Abstract

**Background:**

The nucleotide excision repair (NER) protein, xeroderma pigmentosum C (XPC), participates in recognizing DNA lesions and initiating DNA repair in response to DNA damage. Because mutations in *XPC *cause a high risk of cancer in XP patients, we hypothesized that inherited sequence variations in *XPC *may alter DNA repair and thus susceptibility to cancer.

**Methods:**

In this hospital-based case-control study, we investigated five *XPC *tagging, common single nucleotide polymorphisms (tagging SNPs) in 1,010 patients with newly diagnosed lung cancer and 1,011 matched cancer free controls in a Chinese population.

**Results:**

In individual tagging SNP analysis, we found that rs3731055*AG+AA *variant genotypes were associated with a significantly decreased risk of lung adenocarcinoma [adjusted odds ratio (OR), 0.71; 95% confidence interval (CI), 0.56–0.90] but an increased risk of small cell carcinomas [adjusted OR, 1.79; 95% CI, 1.05–3.07]. Furthermore, we found that haplotype *ACCCA *was associated with a decreased risk of lung adenocarcinoma [OR, 0.78; 95% CI, 0.62–0.97] but an increased risk of small cell carcinomas [OR, 1.68; 95% CI, 1.04–2.71], which reflected the presence of rs3731055*A *allele in this haplotype. Further stratified analysis revealed that the protective effect of rs3731055*AG+AA *on risk of lung adenocarcinoma was more evident among young subjects (age ≤ 60) and never smokers.

**Conclusion:**

These results suggest that inherited sequence variations in *XPC *may modulate risk of lung cancer, especially lung adenocarcinoma, in Chinese populations. However, these findings need to be verified in larger confirmatory studies with more comprehensively selected tagging SNPs.

## Background

The high incidence of lung cancer is a major public health problem worldwide [1]. In China, lung cancer has become the leading cause of cancer-related deaths in both men and women [2]. A number of epidemiological studies have confirmed that approximate 90% of individuals with lung cancer had a direct exposure to tobacco smoke [3], in which some carcinogens can result in DNA damage, leading to genomic instability and malignant transformation of the cell [4]. Nevertheless, only a small fraction of smokers develops lung cancer, suggesting that individual susceptibility may play an important role in the etiology of lung cancer [5].

Lung cancer risk is likely due to an interplay between exposure to etiologic agents and cellular stress response [6]. Under normal conditions, the levels of DNA damage and the capacity of DNA repair systems maintain a dynamic balance; and deficient repair systems can result in either altered apoptosis or unregulated cell growth that leads to carcinogenesis[7]. In humans, DNA damage caused by either ultraviolet light in the sun and carcinogens in cigarette smoke is mainly repaired by the nucleotide excision repair (NER) pathway [8-10]. Considerable evidence suggests that NER capacity is crucial in maintaining normal cell functions, and variations the in DNA repair capacity (DRC) among individuals may contribute to differences in risk of cancers, including lung cancer [11]. The underlying molecular mechanisms of individual variation in cancer susceptibility are thought to be due to genetic polymorphisms, particularly single nucleotide polymorphisms (SNPs) involved in cellular mechanisms, such as DNA repair, that maintain normal cell growth [12]. Therefore, it is likely that inherited sequence variations of the NER genes mayaffect individual susceptibility to cancer as seen in the recessive genetic syndrome xeroderma pigmentosum (XP) [13].

Recently, two studies in Asian populations [14,15] suggest that genetic polymorphisms in the *XPC *gene may be associated with risk of lung cancer, but these studies were either relatively small or the genotyping work did not take into account of all reported SNPs in the *XPC *gene. To further investigate the association between the *XPC *gene and risk of lung cancer in Chinese populations, we took a different approach. Using the *XPC *SNP information available in the National Institute of Environmental Health Sciences (NIEHS) Environmental Genome Project (EGP) SNP database, we identified five representative tagging SNPs that may capture all 29 common (i.e., a minor allele frequency, MAF, ≥ 0.1) SNPs out of 145 reported SNPs [16]. Then, we conducted a large-scale case-control study with 1,010 primary lung cancer patients and 1,011 age and sex frequency-matched cancer-free controls in a Chinese population to evaluate the association between *XPC *genotypes/haplotypes containing variant alleles of these selected tagging SNPs and lung cancer risk.

## Methods

### Study subject

The subject recruitment was described elsewhere [17]. Briefly, all the subject were genetically unrelated ethnic Han Chinese; all patients with primary lung cancer diagnosed between July 2002 and November 2004 according to the National Diagnosis Standard for Lung Cancer were consecutively recruited from four hospitals of three metropolitan cities along the Yangzi River, including the Cancer Hospital of Jiangsu Province, the First Affiliated Hospital of Nanjing Medical University, the Shanghai Cancer Hospital of Fudan University, and the Wuhan Zhongnan Hospital. There were no age, sex, and histology restrictions, but the patients with previous cancer history or unknown conditions of radiotherapy or chemotherapy were excluded. During the study period, a total of 1,299 patients who had confirmed lung cancer by histopathologic diagnosis were recruited from these hospitals, of these patients 1,010 signed inform consents to participate in this study and provided blood samples, resulting in a response rate of 77.8% (1,010/1,299). The cancer-free control subjects came from other clinics departments of the same hospitals during the same period when the cases were recruited. They were outpatients in the clinics of general surgery, gynecology, internal medicine, orthopedics, and otorhinolaryngology and without any tobacco-related diseases, such as emphysema and bronchitis, in the lung. All controls were frequency matched to the cases by age (± 5 years), sex, and residential area (urban or countryside). The response rate in the eligible controls, who signed the informed consent form and donated blood samples, was 81.3% (1,011/1,244).

Epidemiologic data were collected by trained interviewers through a structured questionnaire, which elicited information about sociodemographic characteristics, recent and prior tobacco smoke history, passive smoking, and personal and family history of cancer defined as any reported cancer in the first-degree relatives. Participants who smoked less than one cigarette per day in shorter than one year were defined as nonsmokers; otherwise, they were considered smokers. The smokers' cumulative smoking dose (pack-years) was defined as the number of packs of cigarettes smoked per day multiplied by the number of smoking years. The cut-off point to classify light and heavy smokers was based on the median pack-years of the smokers in the controls. After interview, approximate 5-ml venous blood sample was collected from each participant. The institutional review boards of Nanjing Medical University, Fudan University, and Tongji Medical College of Huazhong University of Science and Technology approved the study protocols.

### Polymorphism selection

The tagging SNPs were selected from the resequencing data of 90 individuals with mixed ethnic background in the NIEHS EGP SNP database [18], base on the following criteria: a) a MAF of at least 0.05 or greater in the promoter region and 0.10 or greater across the whole genomic region, b) putative functional potentials SNPs (i.e., non-synonymous SNPs, promoter SNPs, and SNP at exon-intron boundaries), and c) SNPs that were in linkage disequilibrium (LD) with other SNPs (the threshold of minimal pair-wise r^2 ^= 0.4 due to financial constraints). Based on the first criteria, there were 29 common SNPs in the 145 reported SNPs in the *XPC *gene. We selected the tagging SNPs based on the MAFs of a mixed ethnic group included in the NIEHS EGP SNP database at the time when the HapMap data were not available, in which the MAFs of some SNPs of interest from a subset of Chinese are also included. Although Carlson *et al*. recommended a minimal LD parameter r^2 ^threshold of 0.5 for the tagging SNP selection [19], the 90 USA individuals in the EGP database were a mixed ethnic population, and Asian populations (including Chinese) have lower haplotype diversity and thus higher pairwise LD compared with other populations [20].

Therefore we thought the threshold (r^2 ^= 0.4) in our study might be adequately stringent according to a recent study [17]. Among these 29 common SNPs, we selected five tagging SNPs based on the calculation of pairwise LD: two non-synonymous SNPs (rs2228000, 21151*C *> *T *or A499V and rs2228001, 33512*A *> *C *or K939Q), two common SNPs in the promoter region (rs2607775, 947*C *> *G *and rs3731055, 603*G *> *A*), and one common SNP (rs3729587, 12413*C *> *G*) at an exon-intron boundary.

### XPC genotyping

Genomic DNA was extracted from the whole blood using a DNA blood kit (Gentra Corp., Minnesota, USA) according to the instructions of the manufacturer and stored at -20°C until used. Genotyping was performed by the 5'-nuclease (TaqMan) assay with fluorescent minor groove binding probes [21] at Chinese National Human Genome Center in Shanghai, China. The TaqMan primers and probes were designed by using the Primer Express Oligo Design software v2.0 (ABI PRISM) (available upon request). PCR reactions were performed in 384-well plates, each well containing 5 ng DNA, 2.5 μL 2 × TaqMan Universal PCR Master Mix (Applied Biosystems, Foster City, CA), 0.083 μL 40 × Assay Mix. PCR reaction was initiated at 95°C for 10 min, followed by 20 cycles of 15 s at 92°C and 1 min at 60°C, followed by 30 cycles of 15 seconds at 89°C and 1.5 min at 60°C. After PCR, the fluorescence was detected by the ABI PRISM 7900 HT Sequence Detection System (Applied Biosystems). Two blank (water) controls and two duplicated samples with known genotypes in each 384-well plate were used for the assay quality control. Each SNP that had fluorescence intensity that met the criteria of three clear clusters in two scales generated by the SDS software (ABI) was considered a successful genotype call. As a result, genotype calls failed in 26 (2.6%) controls and 43 (4.2%) cases in the rs3731055 locus, 17 (1.7%) controls and 18 (1.8%) cases in the rs2607775 locus, 78 (7.7%) controls and 74 (7.3%) cases in the rs3729587 locus, 21 (2.1%) controls and 16 (1.6%) cases in the rs2228000 locus and 19 (1.9%) controls and 19 (1.9%) cases in the rs2228000 locus owing to DNA quantity or quality. For quality control, 5% of the samples were re-tested and the concordance was 99.9%.

### Statistical analysis

Differences between the cases and controls in selected demographic variables, including smoking status, smoking quantity (pack-years), andfamily history of cancer were evaluated by the χ^2^test. The paired T-test was also performe d to evaluate the difference in age and smoking quantity (pack-years). The χ^2^test for trend was used to evaluate the increasing levels of smoking (i.e., the trend of never smokers, < 30 pack-years, and > 30 pack-years). The Hardy-Weinberg equilibrium of the alleles at each individual locus was assessed by a goodness-of -fit χ^2 ^test, with one degree of freedom to compare the observed genotypes frequencies with the expected ones among the controls. We also used χ^2^test to evaluate differences in genotypic and allele frequencies. The linkage among SNPs in the *XPC *gene was estimated by the LD coefficient (D') that was calculated by the LDA program [22]. All genotype data of each sample were taken to infer the haplotypes by using the PHASE 2.0 program [23], a software for the reconstruction of haplotypes from the observed genotype data by using a Bayesian statistical approach. We also used the THESIAS program, a software based on the maximum likelihood model described by Tregouet *et al*. [24] and linked with the Stochastic-EM algorithm [25], to estimate the haplotype frequencies and compared the haplotypes with those derived from the PHASE program [23]. The associations between the frequencies of variants in the *XPC *gene and lung cancer risk were estimated by computing odds ratios (ORs) and 95% confidence intervals (CIs) from the logistic regression models with adjustment for age, sex, residential area, family history of cancer, and pack-years of smoking. Potential gene-smoking interaction at a multiplicative scale was also evaluated in the logistic regression analysis and tested by comparing the changes in deviance (-2 log likelihood) between the models for main effects with or without the interaction term. All the statistical analyses were performed with Statistical Analysis System software (v.8.0e; SAS Institute, Cary, NC).

## Results

The primary information of the selected SNPs from different database and the observed genotyping data is shown in Table 1. Although the observed MAFs of all SNPs were very similar between the cases and controls, the observed MAFs of two non-synomynous SNPs (i.e., A499V and K939Q) from the controls (0.32 and 0.36, respectively) were close to that (0.30 and 0.38, respectively) for Chinese obtained in the HapMap database but higher than that (0.24 and 0.34, respectively) from the EGP database. However, the observed MAFs of the other three SNPs, not available in the HapMap database, were dramatically different from those obtained from the EGP database, suggesting that indeed these SNPs may have some ethnic differences in their MAFs. Thus, our original selection of these SNPs from the mixed populations in the EGP database was not optimal and may not represent the LD in Chinese populations.

Epidemiologic data has been described elsewhere [17]. Briefly, the mean age of cases was 60.0 ± 10.8, which was no significant difference with that in controls (59.7 ± 12.0, *P *= 0.61). However, the case group had a higher prevalence of smoking (68.8%) than the controls (52.2%, *P *< 0.001). Furthermore, the cases had higher values of pack-years smoked than the controls (*P *for trend < 0.001); 44.5% of smokers among the cases smoked for ≥ 30 pack-years, whereas this value was only 25.4% among the controls (*P *< 0.0001). The cases were more likely than the controls to report a family history of cancer in their first-degree relatives (17.1% versus 12.8%; *P *= 0.0059). Among the cases, 430 (42.6%) were classified as adenocarcinoma (AC), 335 (33.2%) as squamous cell carcinoma (SCC), 65 (6.4%) as small cell lung carcinoma (SCLC) and 180 (17.8%) as other types, including large cell, mixed cell or undifferentiated carcinomas.

Genotype frequencies of the five selected *XPC *tagging SNPs among cases and controls are shown in Table 2. There was no significant difference between genotype distributions of the control subjects and that expected from the Hardy-Weinberg equilibrium (data not shown). Although the rs3731055 *A *allele frequency was lower among the cases than among the controls (25.1% vs. 27.5%), the difference was not statistically significant (*P *= 0.091), whereas the allele frequencies of other polymorphisms (i.e., rs2607775 *G *allele, rs2228000 *T *allele, rs2228001 *C *allele, and rs3729587 *G *allele) werenon-significantly higher among the cases than among the controls. When lung cancer cases were stratified by tumor histology, rs3731055 genotype distribution among the lung AC was significantly different from that among the controls (*P *= 0.024). Specifically, the rs3731055 *A *allele frequency was lower in the lung AC group (22.9%) but higher in the SCLC group (36.2%), compared to that of the controls (27.5%; *P *= 0.012 and *P *= 0.035, respectively) (Table 2).

The associations between the genotypes of *XPC *tagging SNPs and lung cancer risk are also shown in Table 2, in which all adjusted ORs and 95% CIs were calculated using the common homozygous genotype as the reference group, assuming a recessive genetic model as seen in XP patients [26]. In the individual tagging SNP analysis, the combined rs3731055 *AG+AA *genotype was associated with a significantly decreased risk of all lung cancer, compared with the rs3731055 *GG *genotype (adjusted OR, 0.82; 95% CI, 0.68 – 0.99; *P *= 0.036), but there was no evidence of associations between the genotypes of other tagging SNPs and overall lung cancer risk. When the results were stratified by tumor histology, we found that compared with the rs3731055 *GG *genotype, the combined rs3731055 *AG+AA *genotype was associated with a significantly decrease risk of lung AC (adjusted OR, 0.71; 95% CI, 0.56 – 0.90; *P *= 0.004) but an increase risk of the SCLC group (adjusted OR, 1.79; 95% CI, 1.05 – 3.07; *P *= 0.034).

The results of the haplotype analysis are shown in Table 3, and there were a total of eleven estimated haplotypes out of the 32 (i.e., 2^5^) possible haplotypes in this study population. Compared with the most common haplotype *GCCCC*, haplotype *ACCCA *was associated with a decreased risk of lung AC (OR, 0.78; 95% CI, 0.62 – 0.97; *P *= 0.026) but an increased risk of SCLC (OR, 1.68; 95% CI, 1.04 – 2.71; *P *= 0.032), which is consistent with the results for rs3731055 *A *allele that was present in haplotype *ACCCA*.

We further performed the stratification analysis for the variant rs3731055 genotypes. As show in Table 4, we found that the protective effect of rs3731055 *AG+AA *was more pronounced in young subjects (≤ 60, adjust OR, 0.65; 95% CI, 0.50 – 0.85; *P *= 0.001), non-smokers (adjust OR, 0.74; 95% CI, 0.55 – 0.99; *P *= 0.044) and patients with lung AC (adjusted OR, 0.71; 95% CI, 0.56 – 0.90; *P *= 0.004), whereas these genotypes remained a risk factor for the SCLC group (adjusted OR, 1.79; 95% CI, 1.05 – 3.07; *P *= 0.034).

## Discussion

In this large-scale case-control study, we investigated the associations between five tagging SNPs of the DNA repair gene *XPC *and risk of lung cancer in a Chinese population. Our results showed that the rs3731055 *AG+AA *genotype was associated with a decreased overall risk of lung cancer, especially among young subjects (age ≤ 60 years old), non-smokers, and patients with lung AC, but an increased risk of SCLC. When we evaluated the haplotypes derived from all 5 tagging SNPs, we also found that the haplotype *ACCCA *containing the rs3731055 *A *allele was significantly associated with a decreased risk of lung AC but an increased risk of SCLC. Considering both potential biological functions and use of the tagging SNPs representative of other untyped SNPs, our results may be due to the rs3731055 SNP that is in LD with A499V and K939Q (r^2 ^= 0.17 and 0.21, respectively, in this study population, stronger than 0.025 and 0.040, respectively, obtained from the mixed populations in the NIEHS SNP database), or it is likely that the rs3731055 SNP may be in LD with other untyped disease-causing SNPs. In addition, studies showed that the *XPC *promoter region contains some binding sites of transcription factors, such as p53 [27], AP1, and EGR1 [28]; thus, rs3731055 *G *> *A *change might alter the effect on these protein-DNA interactions. However, the functional relevance of rs3731055 SNP needs further investigations.

XPC is an important damage-recognition protein that recognizes a variety of bulky DNA damage, including UV-induced photolesions and chemical carcinogen-induced DNA adducts, that are repaired by both transcription-coupled and global genome repair processes [29,30]. XPC can also interact with many other important proteins, such as the transcription factor IIH (i.e., TFIIH) [31,32] and the centrisome protein Centrin 2 (CEN2) [33]. In addition to its role in DNA repair, XPC also play an important role in cell-cycle arrest and activation of the p53 pathway [34]. Furthermore, reduced XPC mRNA and protein levels were more frequently observed in both XP heterozygotes [35] and lung cancer patients [36], suggesting that the amount of XPC may modulate susceptibility to cancer.

Although the XPC protein is known to play an important role in the NER pathway, the results of published association studies on *XPC *SNPs and risk of lung cancer remain inconsistent. There are only a few published studies that investigated the role of *XPC *SNPs in the etiology of lung cancer, mostly in Asian populations. For example, Hu *et al*. reported that compared with the 499*CC *(i.e., rs2228000) and 939*AA *(i.e., rs2228001) wild-type homozygotes, subjects carrying 499*CT*+*TT *and 939 *AC*+*CC *respectively had a 1.57-fold and 1.21-fold increased the risk of lung cancer in a Chinese population [14], but this association was not observed in another Chinese study [37]. More recently, Lee *et al*. found that rs3731055*AA *genotype was associated with a 2.1-fold increased risk for lung SCC compared to the rs3731055*GG *genotype in a Korean population of 432 lung cancer patients and 432 healthy controls[15]. These differences in risk associations may be due to different etiology and mechanisms of lung cancer in the study populations with different ethnic background. In a Spanish population of 359 lung cancer patients and 375 healthy controls, Marin *et al*. found that the frequency of XPC PAT+ allele was 45.0% in cases and 39.5% in controls, the difference being statistically significant (*P *= 0.032) [38]. Similarly, Vogel *et al*. [39] also reported that *XPC *Lys939Gln, which is linked with XPC PAT, may be risk factor for lung cancer in another Europe cohort study. In order to verify the association, we also conducted this large-scale study and did not find any significant association on Lys939Gln (37.5% vs. 35.8%). This difference may be due to the different ethnic background or small sample size with limited statistical power.

Some recent studies had shown that mutations in the epidermal growth factor receptor gene, which often took place among the patients with lung AC, were more frequent in never smokers and women in eastern populations, whereas such mutations were more frequent in smokers and men in western populations [40,41]. These observations suggested that the arising incidence of lung AC may be associated with not only environmental risk factors, such as N-nitrasomines or other carcinogens in the air pollutions[42,43], but also genetic susceptibility factors in different ethnic groups and possible different smoking behaviors.

A recent animal study had shown that 100% of *XPC*-deficient mice develop spontaneous lung tumors, the majority of which were adenomas; furthermore, when the mice had *XPC *and *Gadd45a *deleted at the same time, their lung adenomas were progressing to non-small cell lung adenocarcinomas [44]. These results suggested that genetic alterations in *XPC*, in interaction with environmental factors, could result in altered susceptibility to different histological types of lung cancer, particularly in the presence of other genetic susceptibility factors. Indeed, the finding that rs3731055 *AG+AA *genotype or haplotype *ACCCA *were associated with an increased risk of SCLC in the present study suggests that different histopathological types may have different etiologies. Recently, Hollander *et al*. reported that some allelic loss of *XPC *in the lung of mice, coupled with carcinogens such as polycyclic aromatic hydrocarbons, resulted in highly frequent small cell lung cancer and some non-small cell lung cancer [45]. However, the result on SCLC may be due to chance because of the relatively small number of observations in the subgroup of patients with SCLC.

In the present study, we found that the protective effect of rs3731055*AG+AA *genotype was more pronounced among young people (≤ 60 years old), suggesting that such a protective effect may have been diminished because of prolong exposure, as age increased, to N-nitrosomines or other carcinogens When the subjects were divided into three subgroups according to cumulative cigarette consumption (i.e., 0 pack-years, < 30 pack-years, and ≥ 30 pack-years of smoking), we observed that this protective effect was more evident in the never smokers. This result further suggests that cigarette smoking may not be the major pathogenic agent involved in the initiation of lung AC but that some as-yet-unidentified carcinogens may have played a major role in the development of lung AC in this study population. This is consistent with a previous study in which lung AC were more frequent in never smokers than in ever smokers in eastern Asians[41]. However, it is also possi ble that these findings may be due to chance because of the small sample size in the subgroup.

Although the present study was considerably larger than previous studies, it was a hospital-based study that has several limitations. First of all, the participation rate was still relatively low for both cases (77.8%) and controls (81.3%), and about seven percent of DNA samples failed in the genotyping for each locus, which may have increased the probability of selection bias. However, the general demographics and tobacco-exposure information of subjects included in the final analysis were similar with those of people who were excluded, and all lung cancer patients and controls were matched on age, sex, and residential area, which may have minimized the selection bias and confounding factors. Second, because some DNA samples failed in the genotyping, we used the Bayesian statistical method to infer the most probable haplotypes, which may have potential errors. However, the difference in haplotype frequencies between the Stochastic-EM algorithm and the Bayesian method were not significantly different in either cases or controls, increasing the reliability of haplotype estimation. Finally, although we consider both the relevance of biological functions and the representativeness of other untyped SNPs in selecting tagging SNPs of the *XPC *gene, this study may be limited because of excluding some non-synonymous SNPs with low frequencies, which may be more important in the etiology of lung cancer.

## Conclusion

In summary, this study provided further evidence that the *XPC *genotypes and haplotypes may contribute to susceptibility to lung cancer. Because the capture of the untyped SNPs by the selected tagging SNPs was not optimal, some causal SNPs may have been missed in this study. Further larger studies with more comprehensively selected tagging SNPs in Chinese populations are needed to confirm our findings and some mechanistic studies are warranted to investigate the functions of *XPC *SNPs and mechanisms underlying their associations with lung cancer risk.

## Abbreviations

XP, Xeroderma pigmentosum; XPC, Xeroderma pigmentosum C; NER, nucleotide excision repair; DRC, DNA repair capacity; SNPs, single nucleotide polymorphisms; tagging SNPs, tagging single nucleotide polymorphisms; AC, adenocarcinoma; SCC, Squamous cell lung carcinomas; SCLC, Small cell lung carcinomas; OR, odds ratio; CI, confidence interval; TFIIH, basal transcription factor; CEN2, centrisome protein Centrin 2.

## Competing interests

The author(s) declare that they have no competing interests.

## Authors' contributions

YB, LX, XY, and ZH performed the analysis, interpreted the data and drafted the manuscript. TW, DL, QW, HS and LJ contributed to the design of the study and revised the manuscript. WC, XY, JY, FW, MS, HM, and HL participated in data collection, DNA isolation and interpreted the data. LY, WY, YW, and WH participated in SNP genotyping and revised the manuscript. HL, GJ, XH, FC, and YB performed the statistical analysis, interpreted the data and helped to draft the manuscript. JQ, YL, and LJ contributed to data acquisition and interpretation. All authors read and approved the final manuscript.

## Pre-publication history

The pre-publication history for this paper can be accessed here:



## Figures and Tables

**Table 1:** Primary information of selected SNPs of the *XPC *gene

Gene [accession no.] and locus	NCBI rs no.	location	AA^a ^change	Base change	MAF^b ^of cases	MAF^b ^of controls	MAF^b ^in EGP^c^	MAF^b ^in HapMap^d^	% genotyped
*XPC *[NCBI: AY131066] 3p25.1	rs3731055	000603, close to 5'UTR		G > A	0.25	0.27	0.07	---	96.6
	rs2607775	000947, close to 5'UTR		C > G	0.04	0.04	0.35	---	98.3
	rs3729587	012413, intron 5		C > G	0.11	0.11	0.35	---	92.5
	rs2228000	021151, exon 9	A499V	C > T	0.33	0.32	0.24	0.30	98.2
	rs2228001	033512, exon 16	K939Q	A > C	0.38	0.36	0.34	0.38	98.2

^a^AA: amino acid.

^b^MAF: Minor allele frequencies.

^c^EGP: US environmental Genome Project SNP database.

^d^HapMap: For Chinese included in the International HapMap Project.

**Table 2:** Frequency distributions of XPC genotypes and their associations with risk of lung cancer

			Histologic type				Histologic type		
					
*XPC *genotypes	Controls n (%)	Cases n (%)	Adenocarcinoma n (%)	Squamous cell n (%)	Small cell n (%)	All Cases OR (95% C.I.)	Adenocarcinoma OR (95% CI)	Squamous cell OR (95% CI)	Small cell OR (95% CI)
rs3731055									
GG	512 (52.0)	541 (55.9)	247 (60.0)	174 (54.5)	24 (38.7)	1.00	1.00	1.00	1.00
AG	404 (41.0)	366 (37.8)	141 (34.2)	126 (39.4)	31 (50.0)	0.83 (0.69–1.01)	0.71 (0.56–0.92)**	0.90 (0.68–1.20)	1.69 (0.97–2.96)
AA	69 (7.0)	60 (6.3)	24 (5.8)*	20 (6.2)	7 (11.3)	0.76 (0.52–1.11)	0.67 (0.41–1.10)	0.83 (0.47–1.45)	2.44 (0.99–6.02)
AG+AA	473 (48.0)	426 (44.1)	165 (40.0)	146 (45.5)	38 (61.3)	0.82 (0.68–0.99)*	0.71 (0.56–0.90)**	0.86 (0.66–1.13)	1.79 (1.05–3.07)*
A MAF^a^	27.5%	25.1%	22.9%*	25.9%	36.2%*				
rs2607775									
CC	925 (93.1)	913 (92.0)	390 (92.2)	306 (92.7)	58 (90.6)	1.00	1.00	1.00	1.00
CG	65 (6.5)	77 (7.8)	31 (7.3)	24 (7.3)	6 (9.4)	1.16 (0.82–1.66)	1.06 (0.67–1.67)	1.08 (0.64–1.81)	1.16 (0.47–2.88)
GG	4 (0.4)	2 (0.2)	2 (0.5)	0 (0.0)	0 (0.0)	0.78 (0.14–4.34)	1.60 (0.28–9.02)	---	---
CG+GG	69 (6.9)	79 (8.0)	33 (7.8)	24 (7.3)	6 (9.4)	1.15 (0.81–1.62)	1.10 (0.71–1.72)	1.08 (0.64–1.81)	1.16 (0.47–2.88)
G MAF^a^	3.7%	4.1%	4.1%	3.6%	4.7%				
rs2228000									
CC	446 (45.1)	452 (45.5)	184 (43.3)	149 (44.9)	31 (48.4)	1.00	1.00	1.00	1.00
CT	456 (46.1)	435 (43.8)	193 (45.4)	149 (44.9)	25 (39.1)	0.98 (0.81–1.19)	1.05 (0.82–1.35)	1.01 (0.77–1.34)	0.79 (0.46–1.38)
TT	88 (8.8)	107 (10.7)	48 (11.3)	34 (10.2)	8 (12.5)	1.28 (0.93–1.77)	1.42 (0.95–2.13)	1.20 (0.75–1.92)	1.37 (0.59–3.14)
CT+TT	544 (54.9)	542 (54.5)	241 (66.7)	183 (55.1)	33 (51.6)	1.03 (0.86–1.24)	1.11 (0.88–1.40)	1.04 (0.80–1.36)	0.88 (0.53–1.48)
T MAF^a^	31.9%	32.6%	34.0%	32.7%	32.0%				
rs2228001									
AA	404 (40.7)	390 (39.4)	163 (38.3)	129 (39.2)	30 (47.6)	1.00	1.00	1.00	1.00
AC	465 (46.9)	459 (46.3)	198 (46.5)	153 (46.5)	28 (44.4)	1.01 (0.83–1.22)	1.03 (0.80–1.33)	1.04 (0.78–1.38)	0.80 (0.47–1.38)
CC	123 (12.4)	142 (14.3)	65 (15.2)	47 (14.3)	5 (8.0)	1.17 (0.88–1.56)	1.31 (0.92–1.88)	1.14 (0.75–1.72)	0.53 (0.20–1.40)
AC+CC	588 (59.3)	601 (60.6)	263 (61.7)	200 (60.8)	33 (52.8)	1.04 (0.87–1.25)	1.09 (0.86–1.38)	1.06 (0.81–1.39)	0.74 (0.44–1.25)
C MAF^a^	35.8%	37.5%	38.5%	37.5%	30.1%				
rs3729587									
CC	768 (82.3)	756 (80.8)	329 (82.4)	260 (84.1)	47 (81.0)	1.00	1.00	1.00	1.00
CG	130 (14.0)	149 (15.9)	59 (14.8)	42 (13.3)	9 (15.5)	1.14 (0.88–1.48)	1.09 (0.77–1.53)	0.93 (0.62–1.38)	1.09 (0.52–2.32)
GG	35 (3.7)	31 (3.3)	12 (2.8)	8 (2.6)	2 (3.5)	0.90 (0.54–1.50)	0.82 (0.41–1.61)	0.59 (0.26–1.33)	0.86 (0.20–3.78)
CG+GG	165 (17.7)	180 (19.2)	71 (17.6)	50 (15.9)	11 (19.0)	1.09 (0.86–1.39)	1.03 (0.75–1.41)	0.85 (0.59–1.23)	1.04 (0.52–2.08)
G MAF^a^	10.7%	11.3%	10.4%	9.3%	11.2%				

^a ^MAF: minor allele frequency

*P < 0.05; **P < 0.01.

**Table 3:** Associations between frequencies of inferred *XPC *haplotypes and risk of lung cancer

				Histologic types of lung cancer					
				
*XPC *haplotypes^a^	Controls	Cases		Adenocarcinomas		Squamous cell		Small cell	
					
		n (%)	OR (95% CI)	n (%)	OR (95% CI)	n (%)	OR (95% CI)	n (%)	OR (95% CI)
All subjects	2022	2020		860		670		130	
GCCCC	721 (35.6)	737 (36.5)	1.00	328 (38.1)	1.00	243 (36.3)	1.00	35 (26.9)	1.00
GCCTA	615 (30.5)	625 (30.9)	0.99 (0.85–1.16)	278 (32.3)	0.99 (0.82–1.21)	208 (31.0)	1.00 (0.81–1.24)	42 (32.3)	1.41 (0.89–2.23)
ACCCA	453 (22.7)	414 (20.5)	0.89 (0.76–1.06)	160 (18.6)	0.78 (0.62–0.97)*	153 (22.8)	1.00 (0.79–1.27)	37 (28.5)	1.68 (1.04–2.71)*
ACGCA	100 (4.6)	90 (4.4)	0.88 (0.65–1.19)	36 (4.2)	0.79 (0.53–1.18)	24 (3.6)	0.71 (0.45–1.14)	7 (5.4)	1.44 (0.62–3.33)
GGGCA	74 (3.7)	78 (3.9)	1.03 (0.74–1.44)	33 (3.8)	0.98 (0.64–1.51)	22 (3.3)	0.88 (0.54–1.45)	6 (4.6)	1.67 (0.68–4.10)
GCGCC	18 (0.9)	28 (1.4)	1.52 (0.83–2.78)	11 (1.3)	1.34 (0.63–2.88)	7 (1.0)	1.15 (0.48–2.80)	0 (0.0)	---
Others ^b^	41 (2.0)	48 (2.4)	1.15 (0.75–1.76)	14 (1.6)	0.75 (0.40–1.40)	13 (1.9)	0.94 (0.50–1.78)	3 (2.3)	1.51 (0.44–5.11)

^a ^Polymorphic bases were listed in the 5' to 3' order.

^b ^Others including the haplotypes with frequencies of less than 1%.

*P < 0.05a

**Table 4:** Stratification analyses for associations between rs3731055 genotypes and risk of lung cancer by selected variables

	*XPC *rs3731055 genotype					
	
Variables	Case (n = 967)		Control (n = 985)		Adjusted OR (95% CI)	
			
	GG	AG/AA	GG	AG/AA	GG	AG/AA
Total	541 (0.56)	426 (0.44)	512 (0.52)	473 (0.48)	1.00	0.82 (0.68–0.99)*
Age (years)						
≤ 60	282 (0.59)	195 (0.41)	240 (0.49)	245 (0.51)	1.00	0.65 (0.50–0.85)**
> 60	259 (0.53)	231 (0.47)	272 (0.54)	228 (0.46)	1.00	1.03 (0.80–1.34)
Sex						
Male	420 (0.56)	329 (0.44)	382 (0.52)	353 (0.48)	1.00	0.82 (0.66–1.01)
Female	121 (0.55)	97 (0.45)	130 (0.52)	120 (0.48)	1.00	0.86 (0.59–1.24)
Smoke status						
None-smoker	178 (0.60)	119 (0.40)	251 (0.53)	221 (0.47)	1.00	0.74 (0.55–0.99)*
Smoker	363 (0.54)	307 (0.46)	261 (0.51)	252 (0.49)	1.00	0.90 (0.71–1.13)
< 30 pack-years	136 (0.56)	107 (0.44)	136 (0.52)	127 (0.48)	1.00	0.88 (0.61–1.25)
≥ 30 pack-years	227 (0.53)	200 (0.47)	125 (0.50)	125 (0.50)	1.00	0.91 (0.66–1.24)
Family history of cancer						
No	435 (0.54)	363 (0.46)	442 (0.52)	416 (0.48)	1.00	0.84 (0.69–1.03)
Yes	106 (0.63)	63 (0.37)	70 (0.55)	57 (0.45)	1.00	0.71 (0.43–1.18)

^a ^ORs were obtained from a logistic regression model with adjustment for age, sex, residential area, and family history of cancer, and smoking (pack/yr).

*P < 0.05; **P < 0.01.
